# A new species of *Dorstenia* (Moraceae) from southeastern Brazil

**DOI:** 10.3897/phytokeys.12.2772

**Published:** 2012-05-08

**Authors:** Alessandra dos Santos, Sergio Romaniuc Neto

**Affiliations:** 1 Instituto de Botânica, Núcleo de Pesquisa e Curadoria do herbário, avenida Miguel Stéfano, 3687, CEP 04301-902, São Paulo, SP, Brasil

**Keywords:** Atlantic forest, Mantiqueira, Pindamonhangaba

## Abstract

*Dorstenia stellaris* is a new species from southeastern Brazil. This species is endemic to the region and differs from the others by its star shaped coenanthium and cordiform leaves. A description and illustration of this species is presented here. *Dorstenia stellaris* is found in moist and shady places, in small populations within the type locality, thus we recommend its inclusion in the endangered (EN) status of conservation.

## Abstract (es)

*Dorstenia stellaris* é uma espécie nova do sudeste do Brasil. A espécie é endêmica da região e difere-se das demais, pelo cenanto estrelado e folha com base cordada. Neste trabalho foi apresentada descrição e ilustração da espécie. *Dorstenia stellaris* é encontrada em locais úmidos e sombreados, em pequenas populações, somente na localidade típica, desta forma, recomendamos sua categorização como em perigo.

## Introduction

*Dorstenia* was described by Carl Linnaeus (1753), and differs from the other Moraceae genera by the presence of rhizomes, herbaceous and sometimes succulent habit and patelliform inflorescences, the coenanthium. It is the second largest genus in the Moraceae, ca. 105 species distributed through Africa and neotropics, with one species extending into Asia. Brazil holds about 37 species, with the majority of these species Brazilian endemics (Romaniuc Neto et al. 2010).

The great number of species and morphological variation in this genus is reflected by numerous infrageneric groups established by Berg and Hijman (1999). The authors proposed the subdivision of the genus into 9 sections, three of which (*Lecanium*, *Dorstenia* and *Emygdioa*) occur within the neotropical region. *Dorstenia stellaris* is placed in *Lecanium* section, being characterized mostly by showing camephytes and nanophanerophytes species with simple leaves, subulate or foliaceous stipules, and an entire coenanthium.

*Dorstenia* is the only genus in the family that shows an herbaceous habit and its populations often tend to occupy restricted areas with favorable ecological niches. *Dorstenia stellaris* is endemic to Mantiqueira Ridge area and has a restricted distribution, being found only on its type locality, in the municipality of Pindamonhangaba. This species occurs in small populations along moist and shady areas.

## Taxonomic treatment

### Dorstenia stellaris

A. Sant. & Romaniuc sp. nov.

urn:lsid:ipni.org:names:77119223-1

http://species-id.net/wiki/Dorstenia_stellaris

Fig. 1 A–D Fig. 2

#### Diagnosis.

Coenanthium ellipticum, irregulariter stellatum et lamina basis cordiformis differt.

#### Type.

BRASIL. São Paulo, Pindamonhangaba, distrito de Ribeirão Grande, near Fazenda São Sebastião do Ribeirão Grande, 26 Nov. 2011, A. Santos et al. 142 (Holotype: SP!); Brasil. São Paulo, Pindamonhangaba, distrito de Ribeirão Grande, Fazenda São Sebastião do Ribeirão Grande, 30 Mar. 1994, I. Cordeiro et al. 1323 (Paratype: SP!); São Paulo, Pindamonhangaba, distrito de Ribeirão Grande, near Fazenda São Sebastião do Ribeirão Grande, 26 Nov. 2011, A. Santos et al. 143, 144, 145, 146 (Paratype: SP!).

#### Description.

Camephytes 30–70 cm tall; stems aerial, erect, hirsute to tomentose; internodes 1–2.5 cm long.; latex white and abundant. Stipules 1–2 mm long., subulate, narrowly triangular, ciliate, persistent to deciduous, hairs white. Leaves distichous to whorled; blade 8–12 × 3.5–5 cm, membranaceous, apex long acuminate, base cordate, adaxial side scabrous, hairs sparse, white, abaxial side puberulous to hirsute, hairs gathered on the veins; margins entire to denticulate; petiole 2.5–5 cm long., hirsute; brochidodromous venation; 5–6 pairs of secondary veins; tertiary veins scalariform. Coenanthium elliptic, 1–2 cm diam., pateliform, stellate, 3–5 angulate, puberulous; margin with sessile bracts, 0.5–1 mm long., mostly on the angle apex, puberulous, fringes 0.5–0.7 mm alt., greenish to vinaceous; peduncle 1.5–2.5 cm long., puberulous. Staminate flowers distributed through the whole coenanthium; stamens 2; perianth 2 lobed. Pistilate flowers distributed through the whole coenanthium: perianth short lobed, whit apex minutely 2–3 lobed, puberulous; stigma 0.5–1 mm long., slender, white. Drupes elliptical, endocarp smooth to verrucosus; stigmas persistent. Seeds with a flat testa.

**Figure 1.**
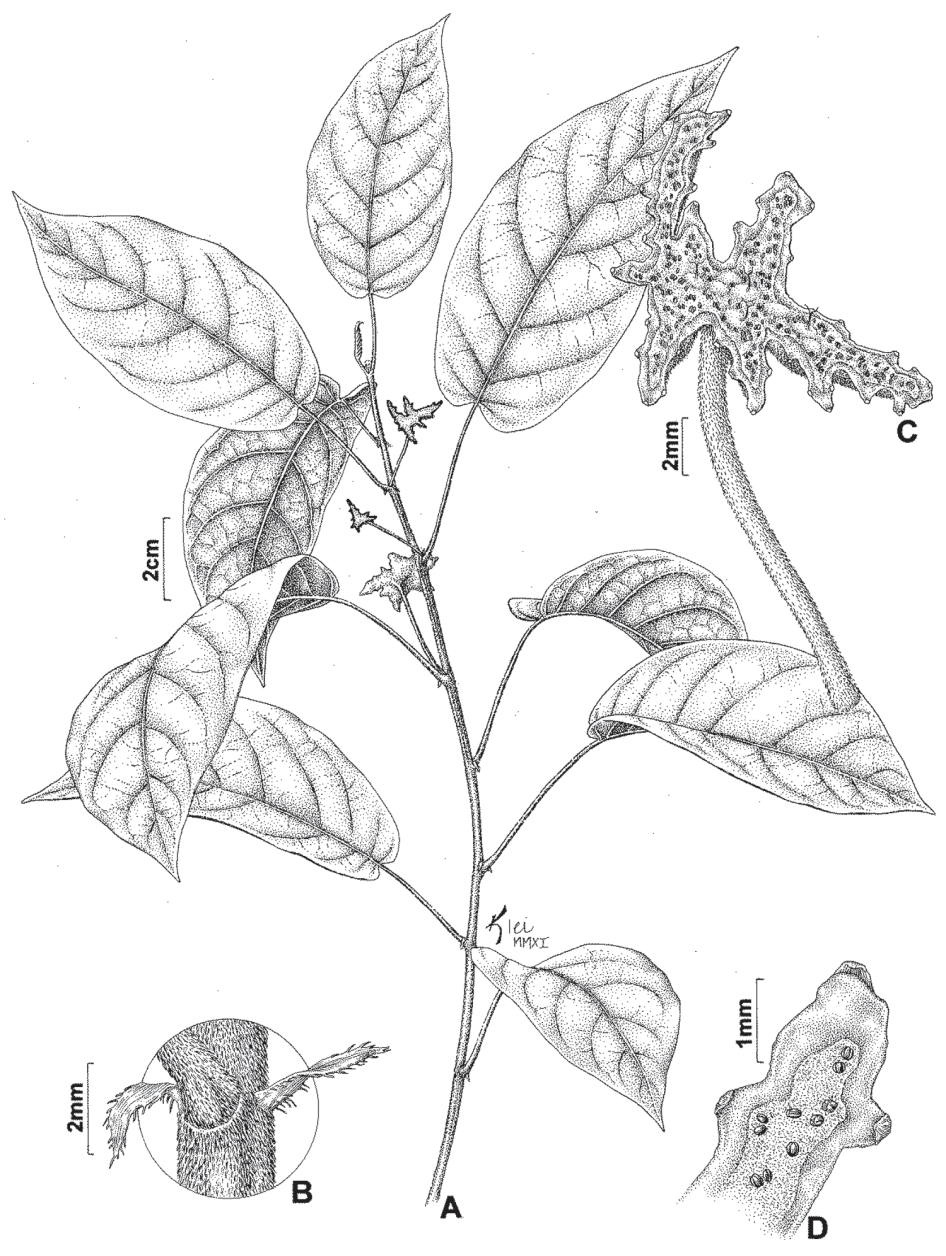
*Dorstenia stellaris* A.Sant. & Romaniuc **A** habit **B** ciliate stipule **C** stellate coenanthium **D** inflorescence, detail of coenanthium fringes and bracts on the angles of the coenanthium margin. (*A. Santos* et al. 142).

**Figure 2.**
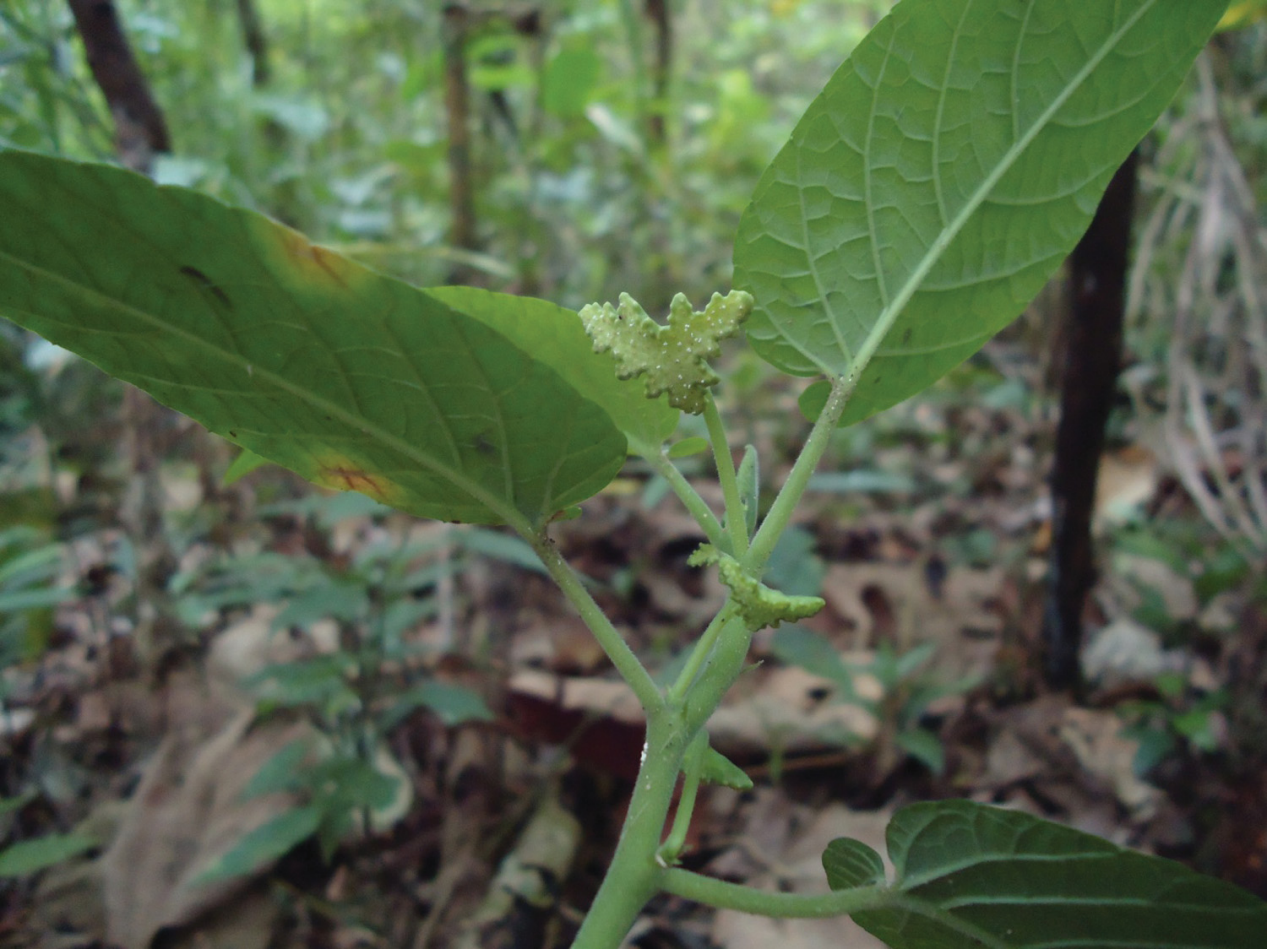
*Dorstenia stellaris* inflorescence, detail of coenanthium (photo: A. Santos 2011).

**Table 1.** Morphological comparison between *Dorstenia* species related to *Dorstenia stellaris*.

Taxa	stellate coenanthium	angulate coenanthium	rounded coenanthium	subulate stipule	base cordate leaves	base<br/> acute leaves
*Dorstenia stellaris*	x			x	x	
*Dorstenia bowmaniana*		x		x		x
*Dorstenia carautae*		x		x		x
*Dorstenia milaneziana*			x	x	x	
*Dorstenia setosa*			x	x	x	

#### Phenology.

Collected with flowers in march and november, and fruits in march.

#### Ecology.

*Dorstenia stellaris* is a camephyte from shady and moist areas within the Atlantic forest, which occur in litter soils, near waterfalls inside forests of the type locality.

#### Similar species.

The angulate shape of *Dorstenia bowmaniana*, and *Dorstenia carautae* coenanthium is similar to that *Dorstenia stellaris*, however, the differs on the strongly irregularly-stellate coenanthium and on the cordate leaves. *Dorstenia milaneziana* and *Dorstenia setosa* are also similar to *Dorstenia stellaris* by the cordate leaves, however, they differ from *Dorstenia stellaris* by the rounded coenanthium. The other species of the *Lecanium* section are mostly orbicular to elliptic coenanthium.

#### Distribution and conservation status.

As *Dorstenia stellaris* is a newly described and very restricted taxon occurring in a non-conserved area, it warrants special attention with regard to its conservation status. This species has only been found within its type locality in small populations. We believe, indeed, that this species is endangered and following IUCN (2011) criteria we recommend its classification within the endangered status of conservation (EN).

#### Etymology.

*stellaris* epithet refers to the irregularly-stellate shape of the coenanthium.

## Supplementary Material

XML Treatment for Dorstenia stellaris
